# Hepatic incidentaloma: An asymptomatic ectopic thyroid tissue

**DOI:** 10.3389/fendo.2022.1066188

**Published:** 2022-12-12

**Authors:** Claudia Di Stefano, Valentina Guarnotta, Maria Barbaccia, Rosario Paratore, Roberta La Monica, Antonio Lo Casto, Massimo Midiri, Salvatore Gruttadauria, Carla Giordano, Pierina Richiusa

**Affiliations:** ^1^ Section of Endocrinology and Diabetology, Health Promotion, Department of Health Promotion, Mother and Child Care, Internal Medicine and Medical Specialties “G. D’Alessandro”, PROMISE, University of Palermo, Palermo, Italy; ^2^ Department of Pathology, Diagnostic and Therapeutic Services, IRCCS ISMETT (Istituto di Ricovero e Cura a Carattere Scientifico-Istituto Mediterraneo per i Trapianti e Terapie ad alta specializzazione)-UPMC (University of Pittsburgh Medical Center), Palermo, Italy; ^3^ Section of “Medicina Nucleare e Terapia Radiometabolica”, La Maddalena, Palermo, Italy; ^4^ Department of Biomedicine, Neurosciences and Advanced Diagnostics, Institute of Radiology, University of Palermo, Palermo, Italy; ^5^ Department for the Treatment and Study of Abdominal Diseases and Abdominal Transplantation, IRCCS-ISMETT (Istituto di Ricovero e Cura a Carattere Scientifico-Istituto Mediterraneo per i Trapianti e Terapie ad alta specializzazione), UPMC (University of Pittsburgh Medical Center), Palermo, Italy; ^6^ Department of General Surgery and Medical-Surgical Specialties, University of Catania, Catania, Italy

**Keywords:** occult thyroid, hepatic thyroid, hyperthyroidism, 131I scintigraphy, hepatic lesion

## Abstract

An ectopic thyroid is a form of thyroid dysgenesis in which the entire thyroid gland or parts of it may be located in another part of the body than the usual place. The most frequent location is the base of the tongue. Although most cases are asymptomatic, symptoms related to tumor size and its relationship with surrounding tissues, hormonal dysfunction, and seldom malignancy may also occur. Here, we describe the case of an asymptomatic woman who was thyroidectomized 19 years previously for a toxic goiter and treated with conventional L-thyroxine therapy, until we enacted a progressive reduction of dosage of the replacement therapy. Incidentally, because of occasional abdomen discomfort, she was hospitalized in our Division of Endocrinology as there was ultrasound evidence of a large mass in the liver dislocating and imprinting the choledochal duct in the pre-pancreatic site, the gallbladder, and the cystic duct, which could not be dissociated from the contiguous hepatic parenchyma and was in very close proximity to the second duodenal portion and the head of the pancreas. Imaging techniques, such as TC, MR, TC/PET, and ^131^I scintigraphy, confirmed the large lesion with a diameter on the axial plane of about 8 × 5.5 cm and a cranio-caudal extension of about 6 cm. The impossibility of surgical debulking and/or radiometabolic ^131^I therapy, in the absence of compression symptoms, led to the multidisciplinary decision of a clinical and instrumental follow-up of this rare lesion.

## Introduction

Ectopic thyroid is a rare abnormality of the embryonic development of the thyroid gland. The estimated frequency is 0.17 per 1,000 patients (1–3). The most common locations are along the midline from the foramen cecum to the mediastinum. Ectopic thyroid tissue usually occurs in the base of the tongue but may also develop in the mediastinum or in the subdiaphragmatic organs (1, 4). Lingual thyroid ectopia is the most frequent form (about 90% of all cases of thyroid ectopia), whereas subdiaphragmatic forms are rather rare. Other distant locations in which ectopic thyroid tissue has been reported include the heart, thymus, esophagus, duodenum, gallbladder, and adrenal glands (4). However, there are rare reports regarding ectopic thyroid in the stomach. Ectopic thyroid tissues in distant sites could be due to aberrant migration or heterotopic differentiation of uncommitted endodermal cells (5–9). According to several reports, a consensual hypothesis for the formation of sub-diaphragmatic thyroid ectopia is failure of migration or intemperate descent of the thyroid anlage (5–7).

Ectopic thyroid is generally more frequent in women and tends to be asymptomatic. Hypothyroidism occurs in about 33% of patients with thyroid ectopy, which is the first cause of congenital hypothyroidism in pediatric age (5). A large percentage of patients with ectopic lingual thyroid without a coexisting eutopic thyroid tissue will develop sub-clinical hypothyroidism that becomes clinically manifest during periods of physiological stress (1, 5, 10). Accessory thyroid tissue may therefore be functional but is generally insufficient for normal function if the main thyroid gland is completely removed (5, 10–12).

## Case description

**Figure 1:**
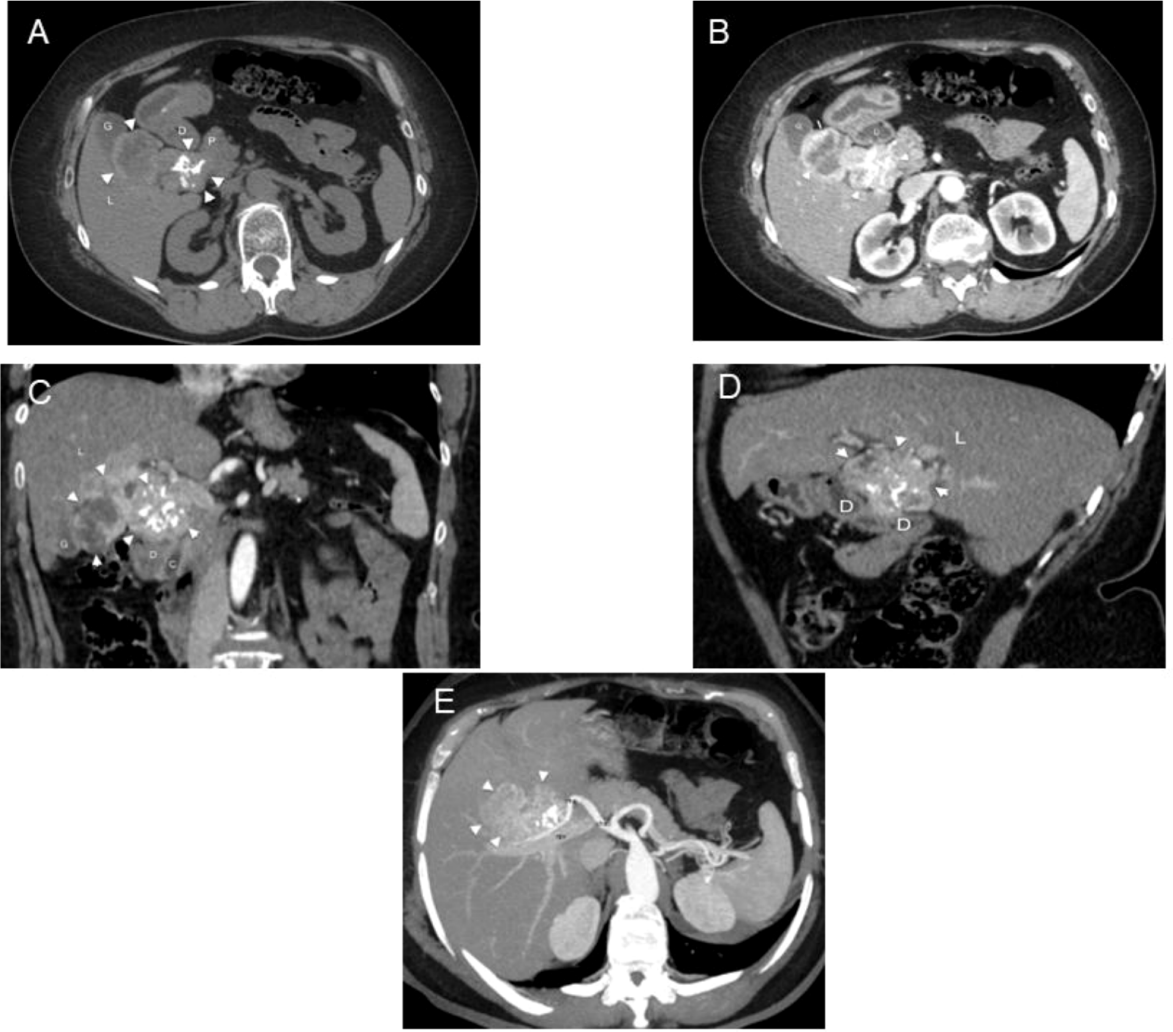
CT in a 65-year-old woman with ectopic thyroid in the hepatic hilum. **(A)** Unenhanced and **(B)** enhanced axial CT images. Dishomogeneous density and partially calcified mass (arrowheads) can be observed in the right hypochondrium between the duodenum (D), liver (L), pancreas (P), and gallbladder (G). Dishomogeneous enhancement of the mass is appreciable in the arterial phase. **(C)** Enhanced coronal CT image. The mass (arrowheads) has a bilobated shape and occupies the hepatic hilum, between the liver (L), gallbladder (G), duodenum (D), pancreas (P), and choledoch (C). **(D)** Enhanced sagittal CT image. The mass (arrowheads) pushes downward the second portion of the duodenum (D), being interposed between it and the liver (L). **(E)** Axial 3D MIP. The mass (arrowheads) is supplied by a branch of the right hepatic artery (rha); cha, common hepatic artery; rpv, right portal branch.

A 65-year-old obese woman was on levothyroxine therapy at the dose of 50 mcg/day (dosage progressively decreased during the last 2 years from 100 mcg/day after surgery). Due to the onset of abdominal pain in the right side and the presence of dark urine, owing to dehydration, the patient had undergone an ultrasound of the abdomen, showing an oval mass of about 7 cm. It had an intensely hyperechoic peripheral portion and a central portion with a dishomogeneous echo structure and was located in close connection with the posterior wall of the gallbladder and with the hepaticum hilum, likely in the duodenal area. Abdomen CT scans with and without contrast (128 slice CT, Somatom Definition AS, Siemens Healthcare, Erlangen, Germany) confirmed the presence in the hepaticum hilum of an extensive tissue portion with irregular margins, unhomogeneously vascularized and characterized by small calcifications in its context, with a diameter on the axial plane of about 8 × 5.5 cm and a cranio-caudal extension of about 6 cm, incorporating the common hepatic duct and imprinting the choledochal duct in the pre-pancreatic site, the gallbladder, and the cystic duct, which could not be dissociated from the contiguous hepatic parenchyma and was in very close proximity to the second duodenal portion and the head of the pancreas (**Figure 1**).

In the opinion of the radiologist, as a first hypothesis, the observations pointed to a heteroplastic lesion of likely pertinence of the biliary tract. Because of the patient’s symptoms (abdominal discomfort and dark urine) and the rise in alkaline phosphatase values, ursodeoxycholic acid therapy at the dose of 450 mg per day was started.

**Figure 2:**
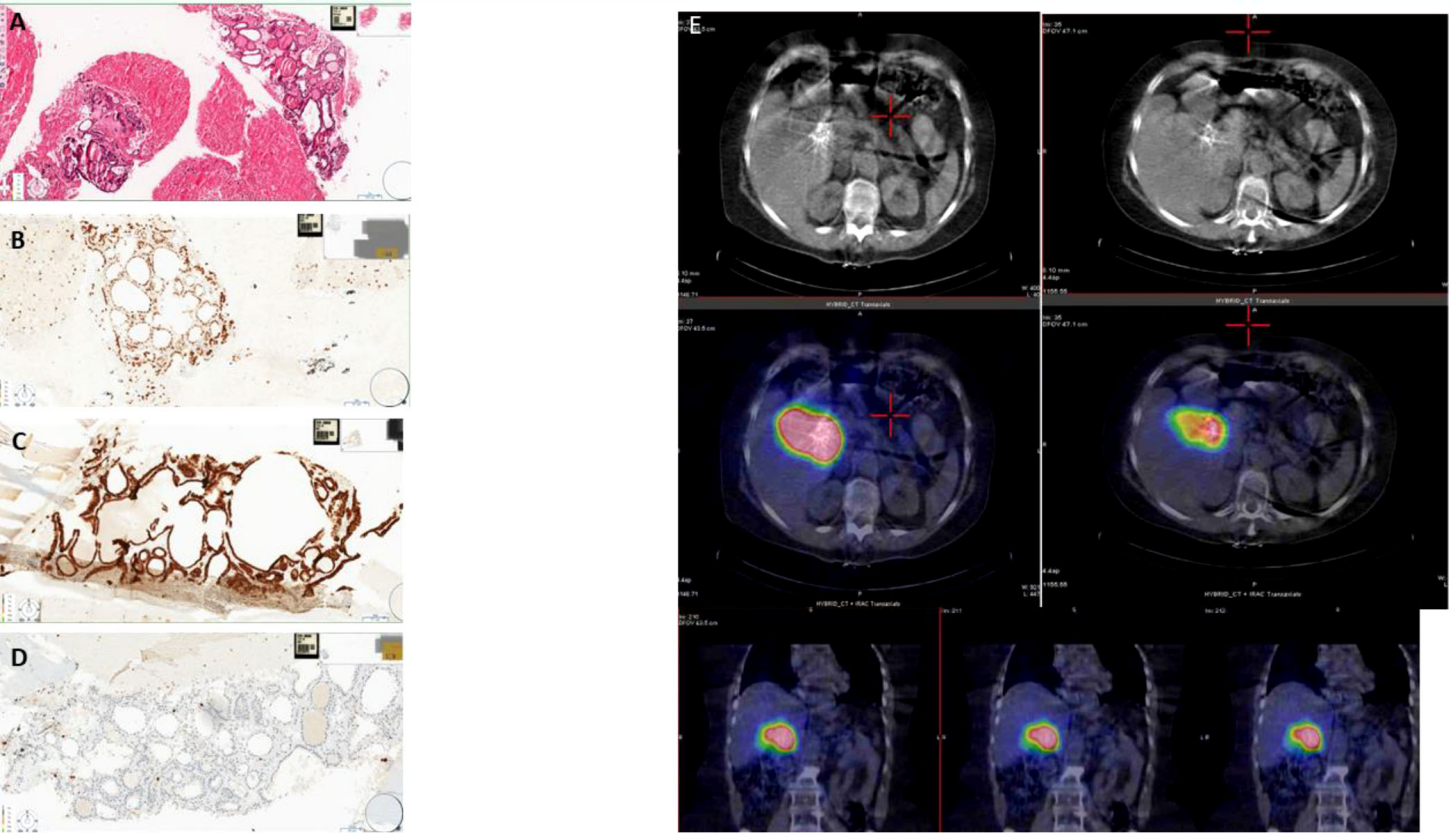
**(A)** Fine-needle aspiration biopsy (section colored in hematoxylin–eosin showing thyroid follicles mixed with red blood cells). **(B)** Fine-needle aspiration biopsy (nuclear positivity for TTF1 in the thyroid follicles). **(C)** Fine-needle aspiration biopsy (membrane positivity for CK7 in the thyroid follicles). **(D)** Fine-needle aspiration biopsy (rare positive nuclei for Ki67). **(E)** CT and SPECT/CT fusion images showing intense ^131^I accumulation in the region of the hepatic hilum site of known ectopic thyroid tissue that contracts with the hepatic parenchyma, its vascular structures, the displaced gallbladder, the duodenum, and the head of the pancreas.

To clarify the nature of the lesion, a fine-needle biopsy of the lesion was performed with ultrasound endoscopy, with a cytological report of a hematoxylin and eosin-stained sample comprising fragments of thyroid tissue, consisting of colloid follicles, whose cells were positive for immunohistochemical staining for TTF1 (**Figures 2A, B**) and PAX8 and CK7 (**Figure 2C**), focally positive for CK19 and negative for synaptophysin and HepPar 1. Ki67 showed a proliferative index <1% (**Figure 2D**). The sample also contained cystic content consisting of macrophages (CD68 positive) and rare fragments of gastrointestinal mucosa.

Thyroid scintigraphy with ^131^I (GE Healthcare Infinia, General Electric Medical Systems, Milwaukee, WI, USA) confirmed the presence of an area of intense accumulation of radioiodine in the upper right quadrant of the abdomen, compatible with ectopic thyroid tissue (**Figure 2E**). Elevated thyroglobulin levels were consistent with the above diagnosis.

Therefore, excluding the suspicion of a lesion originating from intestinal cells, the presence of thyroid tissue in this site required differential diagnosis distinguishing between ectopic thyroid tissue and metastasis from occult thyroid carcinoma.

In addition, in order to exclude the malignant nature of the lesion, the patient underwent total body PET/CT with 18F-FDG, which did not show tracer distribution abnormalities in the exposed regions (not shown).

**Figure 3:**
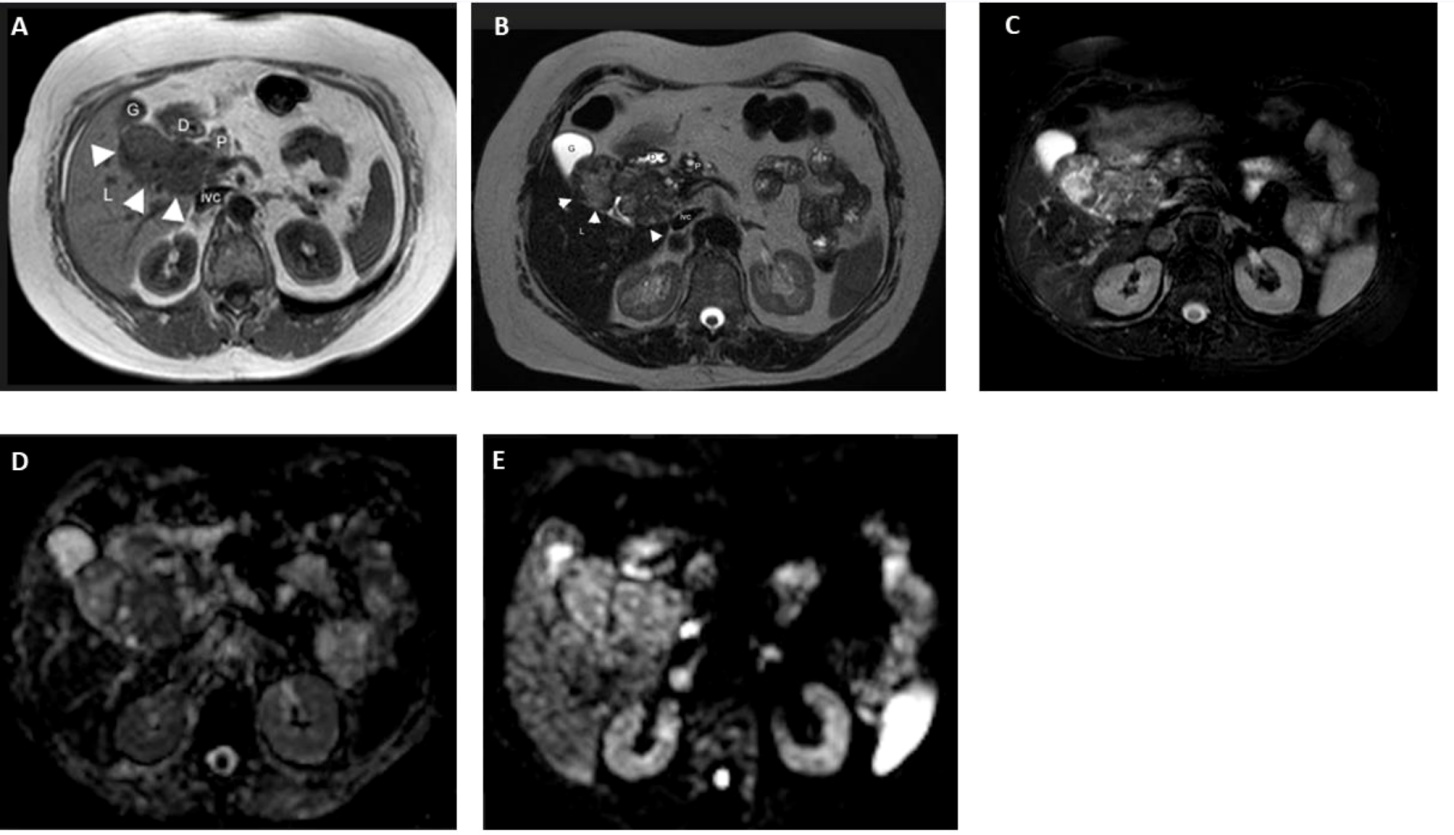
MR in a 65-year-old woman with ectopic thyroid in the hepatic hilum. **(A)** Axial T1 image. Dishomogeneously hypointense (with respect to the liver) mass in the right hypochondrium between the duodenum (D), liver (L), pancreas (P), gallbladder (G), and inferior vena cava (ivc). Axial **(B)** T2 and **(C)** T2 fat-sat images. The mass is dishomogenously hyperintense with respect to the liver. **(D)** DWI B800 image. The mass shows no meaningful restriction. **(E)** ADC image. High ADC in the mass. **(F)** MR-Cholangiography. Common bile duct (cbd) compressed and dislocated by the bilobated mass (m); C, choledocus; G, gallbladder; M (mass); Wirsung (arrowheads).

MRI of the abdomen with and without contrast (1.5 Tesla, Signa HDxt, GE Medical Systems, Milwaukee, WI, USA) confirmed the presence of a polylobulated solid mass slightly hypointense in the T1-weighted sequences and slightly hyperintense in the T2-weighted sequences, with some areas with fluid signal intensity and calcifications in the context, with non-homogeneous post-contrast enhancement, hypointense in the hepatobiliary phase, with a maximum axial size of approximately 7.8 × 4.8 cm located in the gallbladder bed and the hepaticum hilum (**Figure 3**). This mass did not present cleavage planes with the liver, biliary branches and vessels to the hepaticum hilum, gallbladder, duodenum, and head of the pancreas. Due to all the abovementioned clinical and imaging findings, we diagnosed a levothyroxine secreting thyroid ectopia.

We excluded the possibility of any medical treatment due to the size of the lesion, and therefore we focused on the possibility of surgical debulking, through a multidisciplinary evaluation with a team of expert surgeons. At present, the size of the lesion and its extension prevent surgical excision due to the absence, in fact, of clear cleavage planes with the contiguous structures.

In our patient, considering the stability of the lesion at instrumental follow-up and the immunohistochemical pattern, not compatible with malignant thyroid tissue, and having established the impossibility of surgical debulking, in the absence of compression symptoms, we decided on a clinical, biochemical, and instrumental follow-up of the lesion (**Table 1**).

**Table 1:** Clinical and biochemical parameters of the patient from the first observation and during the follow-up.

Clinical parameters	Reference values	Baseline	12 Mo	18 Mo	24 Mo	30 Mo
Levo-thyroxine (mcg/day)		75	75	50	50	50
BMI (kg/m^2^)		30.10	30.10	31.60	30.85	30.06
Waist circumference (cm)		96	99	97	98	97
**Biochemical parameters**						
TSH (mUI/mL)	0.27-4.2	0.01	0.017	0.92	0.41	1.39
FT3 (pg/mL)	2-4.4	2.97	3.03	2.71	2.98	2.84
FT4 (ng/dL)	0.7-1.7	1.16	1.32	0.89	0.87	0.88
Tg (mcg/L)	1.4-78	107	134	ND	191	151.8
AST (U/L)	0-31	13	15	18	16	10
ALT (U/L)	0-31	11	11	16	13	14
Serum bilirubin (mg/dL)	<1.2	0.35	ND	0.98	0.34	0.34
AF (U/L)	35-104	112	121	93	92	121
GGT (U/L)	5-36	32	45	20	30	45
Amylase (U/L)	28-100	45	58	70	62	43
Lipase (U/L)	13-60	23	40	24	21	15

Mo, months; Tg, thyroglobulin; AST, aspartate aminotransferase; ALT, alanine aminotransferase; AF, alkaline phosphatase; GGT, gamma-glutamyl transpeptidase; ND, not detectable.

## Assays

Thyroglobulin, ALT, AST, GGT, alkaline phosphatase, gammaGT, amylase, lipase, and bilirubin were measured in our centralized laboratory with standard methods. TSH, FT4, and FT3 were measured by electrochemiluminescence (ECLIA, Elecsys Insulin, Roche, Milan, Italy).

## Timeline

**Table:** 

December 1999	* Total thyroidectomy
January 2019	Hepatic lesion incidentally discovered at abdominal ultrasound located in close connection with the posterior wall of the gallbladder and with the hepaticum hilum, likely in the duodenal area. CT scan confirmed an extensive tissue portion with irregular margins, with a diameter on the axial plane of about 8 × 5.5 cm and a cranio-caudal extension of about 6 cm, incorporating the common hepatic duct and imprinting the choledochal duct in the pre-pancreatic site, the gallbladder, and the cystic duct, which could not be dissociated from the contiguous hepatic parenchyma and was in very close proximity to the second duodenal portion and the head of the pancreas.
February 2019	Fine-needle biopsy of the abdominal lesion with a cytological report comprising fragments of thyroid tissue, consisting of colloid follicles, whose cells were positive for immunohistochemical staining for TTF1, PAX8, and CK7, focally positive for CK19 and negative for synaptophysin and HepPar 1. Ki67 showed a proliferative index <1%. Thyroid scintigraphy showed the presence of an area of intense accumulation of radioiodine in the upper right quadrant of the abdomen, compatible with ectopic thyroid tissue. MRI scan confirmed the presence of a polylobulated solid mass, with some areas with fluid signal intensity and calcifications in the context, with non-homogeneous post-contrast enhancement, hypointense in the hepatobiliary phase, with a maximum axial size of approximately 7.8 × 4.8 cm located in the gallbladder bed and the hepaticum hilum.
March 2019-Now	Clinical and imaging follow-up

## Discussion

The thyroid is the first endocrine gland to form during embryonic development. During the third or fourth week of gestation, an endodermal diverticulum forms in the ventral part of the pharyngeal intestine which descends in the midline, from the caecum (located between the posterior third and anterior two-thirds of the tongue), positioning itself around the seventh week of gestation in the final location of the gland, anterior to the pretrachea and the larynx. During migration, the gland remains in connection with the floor of the branchial intestine through the thyroglossal duct. The latter undergoes atrophy before the definitive formation of the thyroid gland, approximately between the 6th and 10th weeks of gestation (1, 5, 13). A variety of unexpected locations of thyroid tissues have been reported including gallbladder (14–16), lungs (17–19), duodenum (20), porta hepatis (21), pancreas (22), adrenal glands (23, 24), fallopian tube (25–27), and small intestinal mesentery (28). Here, we report a rare case of thyroid tissue located in the gallbladder bed, accompanied by adenoma and a cyst lined with pseudostratified ciliated columnar epithelium in the region of the gallbladder neck.

According to the well-established embryogenetic theory, at the basis of thyroid ectopy there seems to be an abnormal or aberrant descent of the thyroid sketch during intrauterine life; ectopic tissue can lodge in any site of the normal migration path and may or may not coexist with a eutopic thyroid gland (1, 5, 29).

The most common interpretation is that of abnormal differentiation of the endodermal precursors of the anterior intestine (30). Furthermore, ectopic thyroid tissue could also be explained as a teratoma (5). The majority of the causes are multifactorial ones associated with the embryological process, and recently genetic research has demonstrated that the gene transcription factors TITF-1 (Nkx2-1), Foxe1 (TITF-2), and PAX-8 are essential for thyroid maturation and differentiation. Mutation in these genes may share a connection with abnormal migration of the thyroid (1, 31).

The cases of intra-abdominal ectopic thyroid gland reported in the literature are very limited.

In our case, once the thyroid nature of the lesion was ascertained, any metastasis from occult thyroid carcinoma or the neoplastic degeneration of the ectopic tissue itself was excluded, considering that ectopic thyroid tissue can undergo pathological processes that cannot be differentiated, clinically and histologically, from those affecting normal thyroid (5). At a first observation, it was immediately clear that the daily requirement of LT4 was absolutely underestimated compared with the pro-Kg dose required in a patient undergoing total thyroidectomy (body weight post thyroidectomy 72.5 kg: dose per kg 0.68 mg; body weight at our first observation 76 kg, dose per kg 0.65 mg) (32). This circumstance should have promptly led the clinician to suspect the presence of a levothyroxine-secreting mass.

As regards the therapeutic management of the abdominal lesion, the possibility of treating it with radioiodine 131 was immediately excluded. Therapy with ^131^I (RAI) is indicated for ablative or adjuvant purposes in patients with differentiated thyroid carcinoma undergoing total thyroidectomy aiming at eradicating any residual thyroid and/or microfocus of carcinoma present in the thyroid tissue (33, 34).

Several case histories indicate that hyperthyroidism with large goiter rarely heals after a single administration of ^131^I, so in these cases the primary indication is surgical (34). The size of the lesion therefore plays a role both in the effectiveness of the treatment and in the dose required and consequently as regards various presumable side effects. RAI is generally well tolerated, whether given for a hyperthyroid disorder or for a compressive goiter. However, adverse effects may occur related to 1) thyroid function; 2) size of the thyroid gland; 3) immunological response; and 4) consequences of extrathyroid irradiation (i.e., transient hyperthyroidism, thyroid inflammation, sialadenitis, immunogenic effects, carcinogenesis) (35). If RAI could have been used in our patient, the site and size of the lesion would significantly have influenced the side effects of its use. Indeed, if we translate the common adverse effects related to the use of RAI to the case in question, it would immediately become clear that there would be catastrophic outcomes related to inflammation, for example, of the surrounding tissues (acute hepatitis) or the thyrotoxic storm that could be unleashed irradiating a lesion of this size. For this reason, although interesting, we dropped the idea of RAI treatment.

The rarity of our case is attributable not only to the site but also to the dimensional and bulk characteristics such as not to present cleavage planes with the liver, biliary branches, vessels to the hepaticum hilum, gallbladder, duodenum, and head of the pancreas and thus compromising the possibility of surgical removal. However, it should be taken into account that further episodes of compression of the biliary duct may occur, and in case of huge enlargement of the mass, surgical resection should be undertaken.

## Conclusion

Currently, there are no clear indications on the management of thyroid ectopia. Surgery is generally recommended, notably in the presence of severe obstruction, bleeding, ulceration, refractory hyperthyroidism, severe/local respiratory disorders, and suspected malignancy (1, 5). In other cases, follow-up must be taken into consideration.

## Data availability statement

The original contributions presented in the study are included in the article/supplementary material. Further inquiries can be directed to the corresponding authors.

## Ethics statement

The studies involving human participants were reviewed and approved by Comitato etico di Palermo. The patients/participants provided their written informed consent to participate in this study. Written informed consent was obtained from the individual(s) for the publication of any potentially identifiable images or data included in this article.

## Author contributions

CS, MB, RP, RM, AC, and SG collected the data. CS and VG drafted the manuscript. CG, PR, and MM edited and revised the manuscript. All authors contributed to the article and approved the submitted version.
